# The clinical significance of macrolide resistance in pediatric *Mycoplasma pneumoniae* infection during COVID-19 pandemic

**DOI:** 10.3389/fcimb.2023.1181402

**Published:** 2023-05-12

**Authors:** Ting-ting Jiang, Lin Sun, Tian-yi Wang, Hui Qi, He Tang, Ya-cui Wang, Qian Han, Xiao-qing Shi, Jing Bi, Wei-wei Jiao, A-dong Shen

**Affiliations:** ^1^ Baoding Key Laboratory for Precision Diagnosis and Treatment of Infectious Diseases in Children, Baoding Hospital of Beijing Children’s Hospital, Capital Medical University, Baoding, Hebei, China; ^2^ Key Laboratory of Major Diseases in Children, Ministry of Education, National Key Discipline of Pediatrics (Capital Medical University), National Clinical Research Center for Respiratory Diseases, Beijing Key Laboratory of Pediatric Respiratory Infection Disease, Beijing Pediatric Research Institute, Beijing Children’s Hospital, Capital Medical University, National Center for Children’s Health, Beijing, China

**Keywords:** *Mycoplasma pneumoniae*, drug resistance, children, clinical feature, genotyping

## Abstract

**Background:**

*Mycoplasma pneumoniae* (MP) is a commonly occurring pathogen causing community-acquired pneumonia (CAP) in children. The global prevalence of macrolide-resistant MP (MRMP) infection, especially in Asian regions, is increasing rapidly. However, the prevalence of MRMP and its clinical significance during the COVID-19 pandemic is not clear.

**Methods:**

This study enrolled children with molecularly confirmed macrolide-susceptible MP (MSMP) and MRMP CAP from Beijing Children’s Hospital Baoding Hospital, Capital Medical University between August 2021 and July 2022. The clinical characteristics, laboratory findings, chest imaging presentations, and strain genotypes were compared between patients with MSMP and MRMP CAP.

**Results:**

A total of 520 hospitalized children with MP-CAP were enrolled in the study, with a macrolide resistance rate of 92.7%. Patients with MRMP infection exhibited more severe clinical manifestations (such as dyspnea and pleural effusion) and had a longer hospital stay than the MSMP group. Furthermore, abnormal blood test results (including increased LDH and D-dimer) were more common in the MRMP group (P<0.05). Multilocus variable-number tandem-repeat analysis (MLVA) was performed on 304 samples based on four loci (Mpn13-16), and M3562 and M4572 were the major types, accounting for 74.0% and 16.8% of the strains, respectively. The macrolide resistance rate of M3562 strains was up to 95.1%.

**Conclusion:**

The prevalence of MRMP strains in hospitalized CAP patients was extremely high in the Baoding area, and patients infected with MRMP strains exhibited more severe clinical features and increased LDH and D-dimer. M3562 was the predominant resistant clone.

## Introduction

*Mycoplasma pneumoniae* (MP) is a prevalent pathogen responsible for respiratory tract infections, comprising 10%-30% of community-acquired pneumonia (CAP) cases in children and young adults (Principi et al., 2001; Michelow et al., 2004). Although MP pneumonia is generally mild and self-limited, there have been increasing reports of severe cases, including myocarditis, urticarial, hepatitis, hemolytic anemia, and others (Narita, 2016).

Macrolides are the recommended first-line antibiotics for treating children with MPP (Principi and Esposito, 2013). However, the rapid global emergence of macrolide-resistant MP (MRMP) has become a significant concern. High rates of MRMP have been observed in Asian countries, particularly in China, Japan and Korea, where rates range from 7.7% to 78.5% (Oishi and Ouchi, 2022). The relationship between MRMP and clinical characteristics remains inconsistent. Some studies in Asia have reported increased disease severity in patients infected with MRMP (Chen et al., 2020), while others have found no significant differences in the clinical course between MRMP and macrolide-susceptible MP (MSMP) infections (Waites et al., 2019). Molecular typing of MP is a useful tool for surveillance and outbreak investigation. The Multilocus Variable-Number Tandem-Repeat Analysis (MLVA) method based on five loci (Mpn1, Mpn13-16) was developed in 2009 (Degrange et al., 2009). However, an amended 4-locus MLVA scheme was later proposed due to concerns over the Mpn1 locus’s instability (Sun et al., 2013). Molecular characterization of MP genotypes and macrolide resistance has been widely investigated, with geographic and temporal variations observed.

In late 2019, the emergence of the COVID-19 pandemic drastically affected daily life worldwide. The widespread implementation of non-pharmaceutical interventions (NPIs) has had a significant impact on the transmission of respiratory infections, including MP. However, the prevalence of MRMP and its impact on the clinical manifestations of CAP in children in Baoding during the COVID-19 pandemic have not been well documented. Therefore, this study aimed to investigate the prevalence of macrolide resistance in MP strains isolated from children with CAP in Baoding and to explore the clinical manifestations and genotypes of MRMP.

## Materials and methods

### Study population

The study recruited children under the age of 18 who were admitted to the Baoding Hospital of Beijing Children’s Hospital, Capital Medical University with CAP between August 2021 and July 2022. Diagnosis of CAP was made in accordance with Chinese guidelines and included clinical symptoms and signs consistent with pneumonia, as well as radiographic evidence of consolidation, infiltrate, or pleural effusion (Respiratory Group of Pediatric Branch of Chinese Medical Association, 2015). Patients with immunodeficiency disorders, those receiving immunosuppressive medications, those with a hospital stay of less than one day, and those with incomplete clinical data were excluded. Demographic and clinical information was collected from medical records.

The institutional review board approved the study protocol, and written informed consent was not required since leftover DNA samples from the clinical microbiology laboratory were used.

### Laboratory testing

The suspected CAP patients underwent a battery of routine laboratory tests, which included a complete blood count, serum biochemistry, C-reactive protein (CRP), procalcitonin (PCT), creatine kinase-myocardial band isoenzyme (CKMB), and D-dimer. Oropharyngeal swabs were collected for detection of MP and macrolide resistance-associated mutations using a multiplex real-time PCR assay (Mole, Jiangsu, China) as previously described (Feng X et al., 2016). Briefly, the P1 gene was targeted to confirm the presence of MP, with a probe labelled with VIC. Two point mutations (A2063G and A2064G) located in domain V of the 23S rRNA gene were analyzed to identify macrolide resistance, with a probe labelled with FAM. The sample was considered as MRMP if both VIC and FAM fluorescence signals were detected. Any remaining DNA from MP-positive samples was stored at -80°C for future use.

### MP genotyping

The genotyping of MP was performed using DNA samples extracted directly from oropharyngeal swabs of MP positive patients, as previously described (Dumke and Jacobs, 2011; Wang et al., 2021). Nested PCR was employed to amplify the four highly discriminatory variable-number tandem-repeat (VNTR) loci (Mpn13, Mpn14, Mpn15, Mpn16). The PCR products were subsequently sequenced by Tianyihuiyuan Biotechnology Co. Ltd. to determine the number of repeats for each locus. The MLVA type was assigned as a string of allele numbers in the order of Mpn13 to Mpn16.

### Case definitions

A patient hospitalized with CAP who tested positive for MP *via* PCR was considered to have MP CAP. A MRMP patient was classified as having a positive result for the point mutation test. Severe MPP was defined as a child with MPP exhibiting at least one of the following characteristics according to the Chinese guidelines (Respiratory Group of Pediatric Branch of Chinese Medical Association, 2015): (1) poor general status; (2) increased respiratory rate; (3) cyanosis and dyspnea; (4) infiltration involving multiple lobes or more than two-thirds of the lung; (5) transcutaneous oxygen saturation of 92% or less in room air; (6) extrapulmonary complications.

### Statistical analysis

The demographic information and laboratory test results of the study population were presented as percentages for categorical variables and medians (interquartile range [IQR]) for continuous variables. Statistical analysis was performed using SPSS Statistics version 23 for Windows. Categorical variables were compared using chi-square or Fisher’s exact test, and non-normally distributed continuous variables were compared using the Mann-Whitney test. Statistical significance was set at a two-tailed P value of less than 0.05.

## Result

### Study population

**Figure 1:**
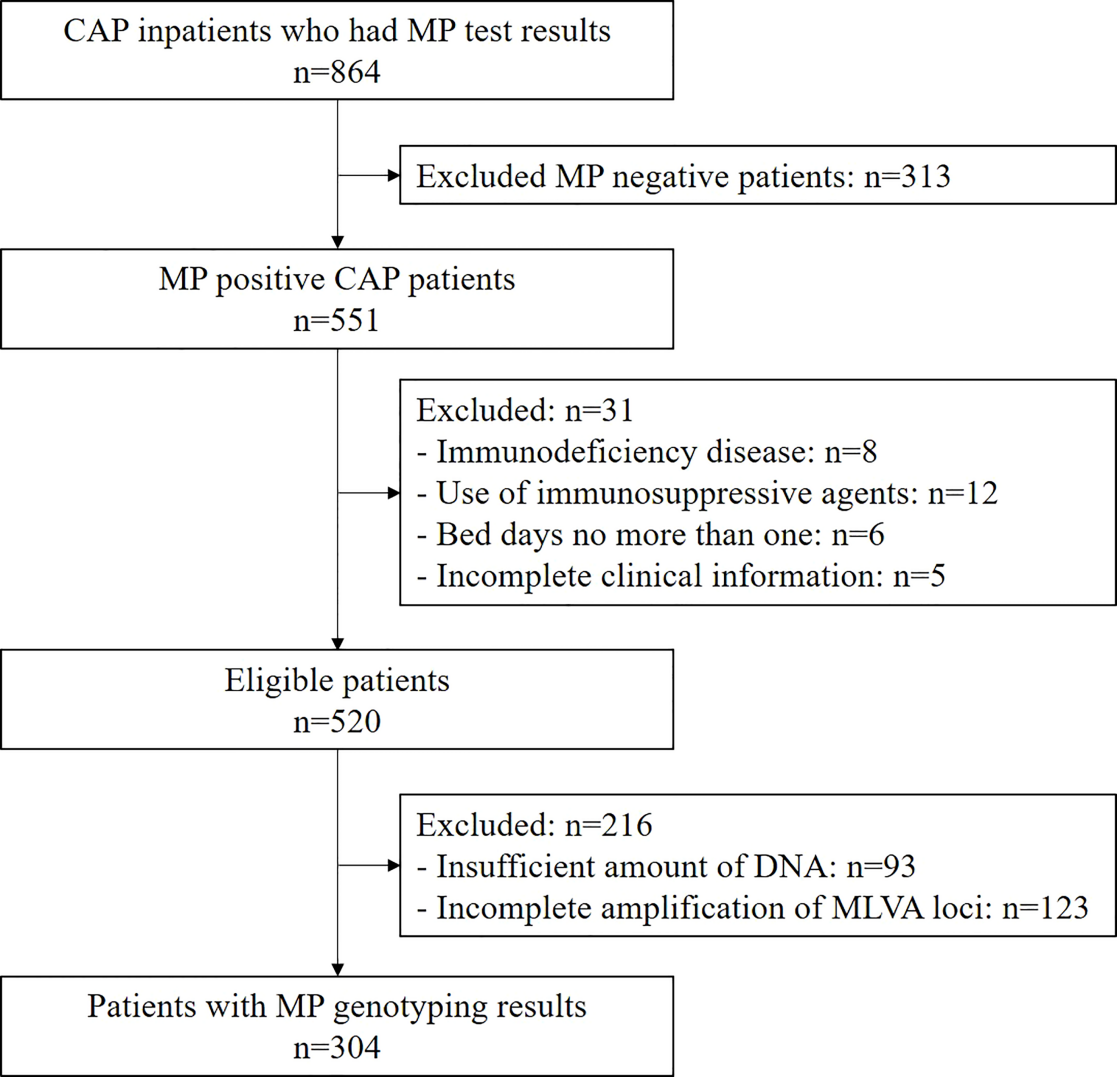
Flow chart of patient enrollment.

Among the 864 children hospitalized with CAP who underwent MP PCR testing, 551 (63.8%) were MP positive. Of these, 520 met the inclusion criteria and were enrolled (**Figure 1**). The median age of the eligible children was 6.63 years (28 days to 17 years), and 50.4% (262/520) were male. The highest prevalence of MP was observed in the 5-9 age group (64.8%, 337/520), followed by the 2-4 age group (15.0%, 78/520), and the 10-18 age group (14.6%, 76/520) (**Table 1**). Severe MP infection was present in 21.2% (110/520) of the enrolled patients.

**Table 1:** Relationship between MP drug resistance and clinical characteristics.

Characteristic	Total (n=520) n (%) or median (IQR)	MRMP patients (n=482), n (%) or median (IQR)	MSMP patients (n=38), n (%) or median (IQR)	*P* value
Basic information				
Age				
0-1	29 (5.6)	24 (5.0)	5 (13.1)	Reference
2-4	78 (15.0)	72 (14.9)	6 (15.8)	0.164
5-9	337 (64.8)	312 (64.7)	25 (65.8)	0.076
10-18	76 (14.6)	74 (15.4)	2 (5.3)	0.017 ^a^
Gender				
Male	262 (50.4)	244 (50.6)	18 (47.4)	0.699
Female	258 (49.6)	238 (49.4)	20 (52.6)	
Clinical manifestations				
Cough	509 (97.9)	473 (98.1)	36 (94.7)	0.189
Fever	490 (94.2)	457 (94.8)	33 (86.8)	0.059
Wheezing	27 (5.2)	24 (5.0)	3 (7.9)	0.437
Dyspnea	71 (13.7)	70 (14.5)	1 (2.6)	0.040 ^a^
Gastrointestinal symptoms	29 (5.6)	29 (6.0)	0 (0.0)	0.257
Skin rash	23 (4.4)	22 (4.6)	1 (2.6)	1
Severe MPP	110 (21.2)	105 (21.8)	5 (13.2)	0.210
Imaging presentations				
Findings				
Infiltrates	386 (74.2)	360 (74.7)	26 (68.4)	0.395
Consolidation	134 (25.8)	122 (25.3)	12 (31.6)	
Involved parts				
Unilateral lung	377 (72.5)	353 (73.2)	24 (63.2)	0.180
Both lungs	143 (27.5)	129 (26.8)	14 (36.8)	
Pleural effusion	126 (24.2)	124 (25.7)	2 (5.3)	0.005 ^a^
Treatment features				
Total days with fever ^b^	8 (6-11)	8 (6-11)	7 (5.75-10)	0.214
Days of hospitalization ^b^	9 (7-12)	9 (7-12)	7 (6-9.25)	0.003 ^a^

MRMP, macrolide resistant *M. pneumoniae*; MSMP, macrolide susceptible *M. pneumoniae*; MPP, *M. pneumoniae* pneumonia; IQR, interquartile range; a: P<0.05; b: non-normal distribution.

### Macrolide susceptibility testing of MP

Of the 520 MP-positive patients, 482 (92.7%) were found to be macrolide resistant and were classified into the MRMP group. The rate of macrolide resistance did not significantly differ among different age groups, ranging from 90.0% in the 0-1 age group to 93.6% in the 4-6 age group (*P*=0.826, **Table 1**). Likewise, there was no significant difference in the rate of macrolide resistance between genders (*P*=0.699, **Table 1**).

### Relationship between MP drug resistance and clinical characteristics

The clinical manifestations were compared between patients with MRMP and MSMP. As shown in **Table 1**, MRMP patients had a significantly higher incidence of dyspnea (*P*=0.040) and were also more likely to experience fever, though the difference was not statistically significant (*P*=0.059). Gastrointestinal symptoms were only observed in MRMP patients (29/482, 6.0%). However, there was no significant difference in other clinical symptoms, including cough, wheezing, rash, and severe MPP, between the two groups.

Regarding radiological findings, MRMP patients did not show a higher incidence of consolidation or bilateral lung involvement compared to MSMP patients, although the differences were not significant (*P*=0.395 and 0.180, respectively) (**Table 1**). Among MP-CAP patients, 126 (24.2%) had pleural effusion, of which 124 patients were in the MRMP group (25.7%, 124/482), indicating that MRMP patients were more likely to develop pleural effusion (*P*=0.005).

The median duration of fever was 8 (IQR 6-11) days for MRMP patients, which was slightly longer than for MSMP patients (7 days, IQR 5.75-10 days). MRMP patients were significantly associated with a longer duration of hospitalization (9 days vs. 7 days, *P*=0.003). Notably, no deaths occurred among the enrolled children.

### Correlation between MP drug resistance and laboratory test indexes

The laboratory findings of children with MRMP and MSMP are presented in **Table 2**. With the exception of LDH and D-dimer, most indicators, such as WBC, platelet, CKMB, ALT, and PCT, did not differ significantly between the two groups. MRMP children had a higher proportion of increased LDH (*P*=0.003) and D-dimer (*P*=0.029) compared to MSMP children. Moreover, a marginal trend towards significance was observed for increased CRP in the MRMP group (60.4% vs. 44.7%, *P*=0.059).

**Table 2:** Correlations between MP drug resistance and laboratory indicators.

Laboratory indicators ^a^	Total (n=520) n (%) or median (IQR)	MRMP patients (n=482), n (%) or median (IQR)	MSMP patients (n=38), n (%) or median (IQR)	*P* value
WBC ^b^				
Normal	423 (81.3)	393 (81.5)	30 (78.9)	Reference
Decreased	18 (3.5)	15 (3.1)	3 (7.9)	0.144
Increased	79 (15.2)	74 (15.3)	5 (13.2)	0.807
Platelet ^c^				
Normal	416 (80.0)	387 (80.3)	29 (76.3)	Reference
Decreased	76 (14.6)	68 (14.1)	8 (21.1)	0.28
Increased	28 (5.4)	27 (5.6)	1 (2.6)	0.71
CRP (mg/L) (<8 mg/L)				
Normal	212 (40.8)	191 (39.6)	21 (55.3)	0.059
Increased	308 (59.2)	291 (60.4)	17 (44.7)	
LDH (110-295 U/L)				
Normal	277 (53.2)	248 (51.5)	29 (76.3)	0.003 ^e^
Increased	243 (46.8)	234 (48.5)	9 (23.7)	
CKMB (0-24 U/L)				
Normal	451 (86.7)	418 (86.7)	33 (86.8)	0.983
Increased	69 (13.3)	64 (13.3)	5 (13.2)	
ALT ^d^				
Normal	451 (86.7)	416 (86.3)	35 (92.1)	Reference
Decreased	7 (1.3)	7 (1.5)	0 (0)	1
Increased	62 (12.0)	59 (12.2)	3 (7.9)	0.605
PCT (<0.25 ng/mL)				
Normal	425 (81.7)	392 (81.3)	33 (86.8)	0.394
Increased	95 (18.3)	90 (18.7)	5 (13.2)	
D-dimer (0-0.256 U/L)				
Normal	227 (43.7)	204 (42.3)	23 (60.5)	0.029 ^e^
Increased	293 (56.3)	278 (57.7)	15 (39.5)	

MRMP, macrolide resistant *M. pneumoniae*; MSMP, macrolide susceptible *M. pneumoniae*; WBC, white cell count; CRP, C reactive protein; LDH, lactate dehydrogenase; CKMB, creatine kinase-myocardial band isoenzyme; PCT, procalcitonin.

a: The reference intervals are listed in parentheses after the test indicators.

b: The reference intervals of WBC: 28 days-<6 months: 4.3-14.2 (×10^9^/L), 6 months-<1 year: 4.8-14.6 (×10^9^/L), 1 year-<2 years: 5.1-14.1 (×10^9^/L), 2 years-<6 years: 4.4-11.9 (×10^9^/L), 6 years-<13 years: 4.3-11.3 (×10^9^/L), 13 years-18 years: 4.1-11.0 (×10^9^/L)(China, 2021a).

c: The reference intervals of platelet: 28 days-<6 months: 183-614 (×10^9^/L) for boys, 203-653 (×10^9^/L) for girls; 6 months-<1 year: 190-579 (×10^9^/L) for boys, 172-601 (×10^9^/L) for girls; 1 year-<2 years: 190-524 (×10^9^/L) for boys, 191-516 (×10^9^/L) for girls; 2 years-<6 years: 188-472 (×10^9^/L) for boys, 187-475 (×10^9^/L) for girls; 6 years-<12 years: 167-453 (×10^9^/L) for boys, 177-446 (×10^9^/L) for girls; 12 years-18 years: 150-407 (×10^9^/L) for boys, 148-399 (×10^9^/L) for girls (National Health Commission of the People's Republic of China, 2021a).

d: The reference intervals of ALT: 28 days-<1 year: 8-71 U/L, 1 year-<2 years: 8-42 U/L, 2 years-<13 years: 7-30 U/L, 13 years-18 years: 7-43 U/L for boys, 6-29 U/L for girls (National Health Commission of the People's Republic of China, 2021b).

e: *P*<0.05 (National Health Commission of the People's Republic of China, 2021b).

### MP genotyping using MLVA

Upon genotyping, 304 samples were found to be viable for analysis, with the remaining 216 samples either lacking sufficient DNA or incomplete MLVA amplification. Among the genotyped samples, nine distinct MLVA types were identified, with the majority of cases exhibiting either the M3562 (225/304, 74.0%) or M4572 (51/304, 16.8%) type. Less common types included M3572 (14/304, 4.6%), M3662 (6/304, 2.0%), and several others (M3472, M4562, M4672, M3462, M4462) that accounted for only 2.6% (8/304) of total cases.

### Relationship between MLVA types and macrolide resistance

**Figure 2:**
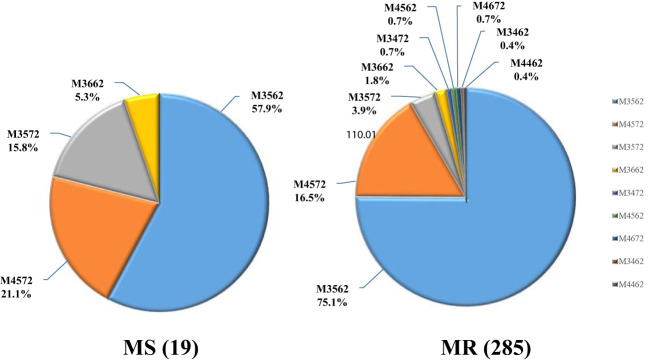
Macrolide resistance in MLVA types.

Of note, the macrolide resistance rate was found to be 93.8% (285/304) in patients with MLVA genotyping results, indicating a predominance of MRMP cases among different MLVA types. The macrolide resistance rates of all MLVA types were high (from 78.6% to 100%). Further analysis revealed a significant association between MLVA type and macrolide resistance, with the M3572 type exhibiting a lower resistance rate (*P*=0.039). The M3572 is a rare MLVA type and has been reported previously (Xiao et al., 2020). Its emergence in Baoding area remained unclear. Interestingly, all MP strains with rare MLVA types (3472/4562/4672/3462/4462) were found to be resistant to macrolide (**Figure 2**; **Table 3**).

**Table 3:** Relationship between genotypes and MP drug resistance.

Genotype	Total (n=304) n (%)	MRMP patients (n=285), n (%)	MSMP patients (n=19), n (%)	*P* value
MLVA type				
3562	225 (74.0)	214 (95.1)	11 (4.9)	reference
4572	51 (16.8)	47 (92.2)	4 (7.8)	0.490
3572	14 (4.6)	11 (78.6)	3 (21.4)	*0.039
3662	6 (2.0)	5 (83.3)	1 (16.7)	0.277
3472/4562/4672/3462/4462	8 (2.6)	8 (100)	0 (0)	1.00
P1 subtype^#^				
1 (4572, 3572)	65 (22.0)	58 (89.2)	7 (10.8)	reference
2 (3562, 3662)	231 (78.0)	219 (94.8)	12 (5.2)	0.147

MLVA, multilocus variable-number tandem-repeat analysis; MRMP, macrolide resistant *M. pneumoniae*; MSMP, macrolide susceptible *M. pneumoniae*; *: *P*<0.05; #: The P1 subtypes were determined based on the relationship between MLVA types and P1 lineages (Sun et al., 2013; Xiao et al., 2020).

Based on the association between MLVA types and P1 lineages reported in previous studies (Sun et al., 2013; Xiao et al., 2020), we determined the P1 subtypes for 296 patients. As presented in **Table 3**, P1-2 subtype was found to be predominant in this study, accounting for 78.0% of the cases. Notably, the prevalence of macrolide resistance was higher in the P1-2 subtype (219/296, 94.8%) compared to P1-1 (58/65, 89.2%). However, no significant correlation was observed between macrolide resitance and P1 subtype (*P*=0.147).

## Discussion

During the COVID-19 pandemic, despite strict implementation of NPIs, we observed a high rate of macrolide resistance in MP-positive pediatric patients (92.7%). As reported in previous studies, MRMP was associated with dyspnea, pleural effusion, longer hospital stays, and abnormal blood test results, including increased LDH and D-dimer. The MLVA type 3562 was the most prevalent (74.0%) among the genotyped samples, with a very high macrolide resistance rate (95.1%).

Macrolide resistance of MP has been increasing globally, with a particularly severe situation in Asian countries (Oishi and Ouchi, 2022). In China, previous studies have reported that more than 90% of MP infections were caused by macrolide-resistant strains (Xin et al., 2009; Zhou et al., 2015). However, a recent prospective, multicenter, population-based surveillance study among Chinese children during the COVID-19 pandemic (2020.1-2021.6) reported a remarkable decrease in MP infections and a decrease in the rate of MRMP to 7.7% (Chen et al., 2022). In contrast, our study found a high rate of macrolide resistance (92.7%) among children with CAP during the COVID-19 pandemic. Possible reasons for the difference in rates of macrolide resistance could be attributed to several factors. Firstly, the enrolled patients in our study were CAP inpatients, while Chen et al. focused on outpatient children with mild respiratory tract infections. Macrolide resistance rates might be higher among inpatients or patients with pneumonia (Guo et al., 2019; Katsukawa et al., 2019). Secondly, macrolide therapy has been shown to promote the development of macrolide resistance (Suzuki et al., 2017). In the study by Chen et al., only 17.6% of the outpatients had prior macrolide prescriptions, while almost all inpatients in our study received macrolide therapy prior to hospitalization. Thirdly, the primary macrolide resistance of MP was higher in the Baoding area, which was similar to data from adjacent cities (93.9% in Beijing in 2020, 90.0% in Shijiazhuang in 2015-2016) (Wang et al., 2016; Wang et al., 2022). Fourthly, the selective pressure for the development of drug resistance may also result from the excessive or inappropriate use of macrolides. It has been reported that the overall rate of antibiotic use in children with CAP in China was 89.08%, with macrolides being the most commonly used antibiotics as the first choice for the treatment of MP-caused CAP (Wei et al., 2019).

MP infection was most commonly observed in the 5-9 age group, and the prevalence of MRMP infection was higher among older children. This is in line with previous findings that school-aged children, who come into contact with more people and are often in closed spaces, are more susceptible to the rapid spread of pathogens (Guo et al., 2019). We conducted further investigations to examine the relationship between macrolide resistance of MP and clinical characteristics. Our findings revealed a significantly higher incidence of dyspnea among children infected with MRMP (*P*=0.040), with a similar trend observed for fever (*P*=0.059). Gastrointestinal symptoms were only observed in MRMP patients (6.0%). Previous studies from Italy and various regions in China have also reported a trend towards higher rates of dyspnea, fever, and extrapulmonary symptoms in MRMP patients (Cardinale et al., 2013; Ma et al., 2014; Yang et al., 2019). However, they did not observe any significant differences in clinical features among MRMP and MSMP patients. The possible reasons for this finding could be the relatively small sample size in previous studies, with the total number of patients with dyspnea being less than 60, or the huge disparity in the number of MRMP and MSMP patients, with MRMP patients being predominant in most Chinese reports while MSMP patients being more common in Europe (Oishi and Ouchi, 2022).

The presence of pleural effusion in MP pneumonia may indicate the severity and prolonged lesions on chest radiography, and it is one of the most common reasons for transfer to tertiary hospitals in patients with MP pneumonia. In this study, pleural effusion was observed in 24.2% of children with MP-CAP, which was slightly higher than previous reports (16%-20.7%) (Fine et al., 1970; Miyashita et al., 2009; Kim et al., 2021). Further analysis revealed that pleural effusion occurred significantly more frequently in the MRMP group (*P*=0.005). While Kim et al. reported that pleural effusion was not associated with macrolide resistance, all 24 patients with pleural effusion in their study belonged to the MRMP group, which is consistent with our findings.

The length of hospital stay was longer in MRMP patients than in MSMP patients (*P*=0.003). This is in line with a recent systematic review, which found that MRMP infections were associated with prolonged hospitalization compared with MSMP infections (*P*<0.001). Although macrolide-resistant strains were associated with more pleural effusion and longer hospital stays, there was no significant correlation between macrolide resistance and severe MPP (*P*=0.210).

We compared common laboratory indicators between CAP patients with MRMP infection and MSMP infection. In accordance with the new clinical biochemistry and blood cell analysis standards for children implemented in October 2021, reference intervals for each indicator vary by age group. Therefore, we used classification indices instead of absolute numbers. As a result, more MRMP patients were found to have elevated LDH and D-dimer levels (*P*<0.05). Previous studies have suggested that serum LDH is a biomarker for predicting refractory MPP and evaluating the need for corticosteroid therapy during early hospitalization (Inamura et al., 2014; Lu et al., 2015; Huang et al., 2021). Similarly, higher D-dimer levels have been associated with more severe clinical manifestations and longer treatment durations (Huang et al., 2021; Zheng et al., 2021). Our findings suggest that elevated levels of LDH and D-dimer are significantly greater in CAP patients with MRMP infection, indicating the severity of disease caused by MRMP. Additionally, we observed a trend towards significance for increased CRP in the MRMP group (*P*=0.059). However, previous studies have typically used absolute numbers for comparison, and no association has been found between CRP and MRMP (Chen et al., 2020).

In this study, we successfully obtained MLVA genotype data of 304 MP strains in specimens and categorized them into nine types. The predominant type was M3562 (74.0%), followed by M4572 (16.8%). However, the most prevalent type reported in Beijing, Taiwan, and Spain was M4572 (79.3%, 73.2%, and 50.1%, respectively), suggesting geographical differences (Rivaya et al., 2020; Wang et al., 2021; Wu et al., 2021). The macrolide resistance rates of M3562 and M4572 were 95.1% and 92.2%, respectively. Previous studies have suggested that the spread of MP appears to be polyclonal, associated with multiple genotypes (Pereyre et al., 2012; Suzuki et al., 2019). Interestingly, Wang et al. found that the prevalence of M3562 increased from 11.63% to 24.67% during 2016-2019 in northern China, with a drastic increase in the macrolide resistance rate from 60% to 93.48% (Wang et al., 2021). This suggests that expansion of drug-resistant MP strains with specific genotype may play an important role in the high rate of macrolide resistance. It appears that the high prevalence of M3562 in this study is the result of drug-resistant genotype expansion. We derived P1 subtypes based on the relationship between MLVA type and P1 lineage and found that MR was more common in the P1-2 subtype, which is inconsistent with previous reports showing that most MRMP strains harbored the P1-1 subtype (Katsukawa et al., 2019; Xiao et al., 2020). However, this may be due to the predominant prevalence of M3562, which belongs to the P1-2 subtype and is highly drug-resistant. Continuous monitoring of MP genotypes will be necessary to understand the epidemiological trends.

Several limitations of our study should be noted. Firstly, the study was conducted in a single center, which may limit the generalizability of our findings to other populations. Secondly, MP detection was not performed for all CAP patients during the study period, which may introduce selection bias. Thirdly, mutations associated with macrolide resistance other than 23S rRNA A2063G and A2064G were not included in this study, which may underestimate the prevalence of macrolide resistance. Fourthly, not all MP-positive samples could be genotyped due to insufficient amounts of DNA or incomplete amplification of MLVA loci, which may have led to bias in the results. Fifthly, the low number of MSMP patients included in our study (compared to MRMP patients) may also have introduced bias, even though we have statistically processed the data. Finally, as with all observational studies, we cannot infer causality, and further studies are needed to confirm our findings.

In conclusion, our study highlights the severity of macrolide resistance in Baoding during the COVID-19 pandemic. Our findings suggest that MRMP infection is associated with more severe clinical manifestations and increased levels of LDH and D-dimer. Additionally, our results indicate that the M3562 clone is the prevailing macrolide-resistant strain in Baoding. Based on our findings, substitution therapy using tetracycline and quinolone should be considered for the treatment of MRMP infections. Furthermore, continuous monitoring of drug resistance and genotypes is recommended to guide treatment strategies and inform public health policies in children.

## Data availability statement

The raw data supporting the conclusions of this article will be made available by the authors, without undue reservation.

## Ethics statement

The studies involving human participants were reviewed and approved by Ethics committee of Baoding hospital of Beijing Children’s Hospital, Capital Medical University. Written informed consent from the participants’ legal guardian/next of kin was not required to participate in this study in accordance with the national legislation and the institutional requirements.

## Author contributions

A-DS, JB, W-WJ were responsible for study design. T-TJ, HQ, T-YW, QH, X-QS collected and organized data. T-TJ, T-YW and Y-CW did the genotyping. T-TJ, LS, HT, W-WJ, A-DS analyzed the data. T-TJ, LS, W-WJ wrote the manuscript, JB and A-DS revised the manuscript. JB and A-DS provided research funds. All authors contributed to the article and approved the submitted version.

## References

[B1] Cardinale F. Chironna M. Chinellato I. Principi N. Esposito S. (2013). Clinical relevance of *Mycoplasma pneumoniae* macrolide resistance in children. J. Clin. Microbiol. 51, 723–724. doi: 10.1128/JCM.02840-12 23224091 PMC3553869

[B2] Chen Y. C. Hsu W. Y. Chang T. H. (2020). Macrolide-resistant *Mycoplasma pneumoniae* infections in pediatric community-acquired pneumonia. Emerg. Infect. Dis. 26, 1382–1391. doi: 10.3201/eid2607.200017 32568052 PMC7323531

[B3] Chen J. Zhang J. Lu Z. Chen Y. Huang S. Li H.. (2022). *Mycoplasma pneumoniae* among Chinese outpatient children with mild respiratory tract infections during the coronavirus disease 2019 pandemic. Microbiol. Spectr. 10, e0155021. doi: 10.1128/spectrum.01550-21 35138173 PMC8826743

[B4] Degrange S. Cazanave C. Charron A. Renaudin H. Bebear C. Bebear C. M. (2009). Development of multiple-locus variable-number tandem-repeat analysis for molecular typing of *Mycoplasma pneumoniae* . J. Clin. Microbiol. 47 (4), 914–923. doi: 10.1128/JCM.01935-08 19204097 PMC2668363

[B5] Dumke R. Jacobs E. (2011). Culture-independent multi-locus variable-number tandem-repeat analysis (MLVA) of *Mycoplasma pneumoniae* . J. Clin. Microbiol. 86 (3), 393–396. doi: 10.1016/j.mimet.2011.06.008 21704086

[B6] Feng X. L. Q. Sun L. Jiao W. Xu B. Yin J. Guo Y.. (2016). The clinical characteristics of macrolide-resistant *Mycoplasma pneumoniae* pneumonia in children: a case-control study. Chin. J. Evid. Based Pediatr. 11, 357–360. doi: 10.3969/j.issn.1673-5501.2016.05.008

[B7] Fine N. L. Smith L. R. Sheedy P. F. (1970). Frequency of pleural effusions in mycoplasma and viral pneumonias. New Engl. J. Med. 283, 790–793. doi: 10.1056/NEJM197010082831505 5456236

[B8] Guo D. X. Hu W. J. Wei R. Wang H. Xu B. P. Zhou W.. (2019). Epidemiology and mechanism of drug resistance of *Mycoplasma pneumoniae* in Beijing, China: a multicenter study. Bosn J. Basic Med. Sci. 19, 288–296. doi: 10.17305/bjbms.2019.4053 30878034 PMC6716100

[B9] Huang X. Li D. Liu F. Zhao D. Zhu Y. Tang H. (2021). Clinical significance of d-dimer levels in refractory *Mycoplasma pneumoniae* pneumonia. BMC Infect. Dis. 21, 14. doi: 10.1186/s12879-020-05700-5 33407216 PMC7787414

[B10] Inamura N. Miyashita N. Hasegawa S. Kato A. Fukuda Y. Saitoh A.. (2014). Management of refractory *Mycoplasma pneumoniae* pneumonia: utility of measuring serum lactate dehydrogenase level. J. Infect. Chemother. 20, 270–273. doi: 10.1016/j.jiac.2014.01.001 24486173

[B11] Katsukawa C. Kenri T. Shibayama K. Takahashi K. (2019). Genetic characterization of mycoplasma pneumoniae isolated in Osaka between 2011 and 2017: decreased detection rate of macrolide-resistance and increase of p1 gene type 2 lineage strains. PloS One 14 (1), e0209938. doi: 10.1371/journal.pone.0209938 30682029 PMC6347185

[B12] Kim S. H. Lee E. Song E. S. Lee Y. Y. (2021). Clinical significance of pleural effusion in *Mycoplasma pneumoniae* pneumonia in children. Pathogens 10, 1075. doi: 10.3390/pathogens10091075 34578108 PMC8469935

[B13] Lu A. Wang C. Zhang X. Wang L. Qian L. (2015). Lactate dehydrogenase as a biomarker for prediction of refractory *Mycoplasma pneumoniae* pneumonia in children. Respir. Care 60, 1469–1475. doi: 10.4187/respcare.03920 26060318

[B14] Ma Z. Zheng Y. Deng J. Ma X. Liu H. (2014). Characterization of macrolide resistance of *Mycoplasma pneumoniae* in children in shenzhen, China. Pediatr. Pulmonol 49, 695–700. doi: 10.1002/ppul.22851 23861188

[B15] Michelow I. C. Olsen K. Lozano J. Rollins N. K. Duffy L. B. Ziegler T.. (2004). Epidemiology and clinical characteristics of community-acquired pneumonia in hospitalized children. Pediatrics 113, 701–707. doi: 10.1542/peds.113.4.701 15060215

[B16] Miyashita N. S. Sugiu T. Kawai Y. Yamaguchi T. Ouchi K. (2009). Radiographic features of *Mycoplasma pneumoniae* pneumonia: differential diagnosis and performance timing. BMC Med. Imaging 9, 7. doi: 10.1186/1471-2342-9-7 19400968 PMC2680832

[B17] Narita M. (2016). Classification of extrapulmonary manifestations due to *Mycoplasma pneumoniae* infection on the basis of possible pathogenesis. Front. Microbiol. 7, 23. doi: 10.3389/fmicb.2016.00023 26858701 PMC4729911

[B18] National Health Commission of the People's Republic of China. (2021a). “Health industry standard of the people’s republic of China- reference intervals of blood cell analysis for children”, (WS/T), 779–2021.

[B19] National Health Commission of the People's Republic of China. (2021b). “Health industry standard of the People's Republic of China-Reference intervals of clinical biochemistry tests commonly used for children” (WS/T), 780–2021.

[B20] Oishi T. Ouchi K. (2022). Recent trends in the epidemiology, diagnosis, and treatment of macrolide-resistant *Mycoplasma pneumoniae* . J. Clin. Med. 11, 1782. doi: 10.3390/jcm11071782 35407390 PMC8999570

[B21] Pereyre S. Charron A. Hidalgo-Grass C. Touati A. Moses A. E. Nir-Paz R.. (2012). The spread of *Mycoplasma pneumoniae* is polyclonal in both an endemic setting in France and in an epidemic setting in Israel. PloS One 7, e38585. doi: 10.1371/journal.pone.0038585 22701675 PMC3368914

[B22] Principi N. Esposito S. (2013). Macrolide-resistant *Mycoplasma pneumoniae*: its role in respiratory infection. J. Antimicrob. Chemother. 68, 506–511. doi: 10.1093/jac/dks457 23169891

[B23] Principi N. Esposito S. Blasi F. Allegra L. Mowgli Study G. (2001). Role of *Mycoplasma pneumoniae* and *Chlamydia pneumoniae* in children with community-acquired lower respiratory tract infections. Clin. Infect. Dis. 32, 1281–1289. doi: 10.1086/319981 11303262

[B24] Respiratory Group of Pediatric Branch of Chinese Medical Association (2015). Guidelines for the management of community acquired pneumonia in children, (2013 Revision). Chin. J. Pediatr. 51, 745–752. doi: 10.3760/cma.j.issn.0578-1310.2013.10.006 24406226

[B25] Rivaya B. Jordana-Lluch E. Fernandez-Rivas G. Molinos S. Campos R. Mendez-Hernandez M.. (2020). Macrolide resistance and molecular typing of *Mycoplasma pneumoniae* infections during a 4 year period in Spain. J. Antimicrob. Chemother. 75, 2752–2759. doi: 10.1093/jac/dkaa256 32653897 PMC7678890

[B26] Sun H. Xue G. Yan C. Li S. Cao L. Yuan Y.. (2013). Multiple-locus variable-number tandem-repeat analysis of *Mycoplasma pneumoniae* clinical specimens and proposal for amendment of MLVA nomenclature. PloS One 8 (5), e64607. doi: 10.1371/journal.pone.0064607 23737989 PMC3667773

[B27] Suzuki Y. Seto J. Shimotai Y. Itagaki T. Katsushima Y. Katsushima F.. (2019). Polyclonal spread of multiple genotypes of *Mycoplasma pneumoniae* in semi-closed settings in yamagata, Japan. J. Med. Microbiol. 68, 785–790. doi: 10.1099/jmm.0.000969 30932805

[B28] Suzuki Y. Shimotai Y. Itagaki T. Seto J. Ikeda T. Yahagi K.. (2017). Development of macrolide resistance-associated mutations after macrolide treatment in children infected with *Mycoplasma pneumoniae* . J. Med. Microbiol. 66, 1531–1538. doi: 10.1099/jmm.0.000582 28984229

[B29] Waites K. B. Ratliff A. Crabb D. M. Xiao L. Qin X. Selvarangan R.. (2019). Macrolide-resistant *Mycoplasma pneumoniae* in the united states as determined from a national surveillance program. J. Clin. Microbiol. 57, e00968–19. doi: 10.1128/JCM.00968-19 31484701 PMC6813023

[B30] Wang X. Li M. Luo M. Luo Q. Kang L. Xie H.. (2022). *Mycoplasma pneumoniae* triggers pneumonia epidemic in autumn and winter in Beijing: a multicentre, population-based epidemiological study between 2015 and 2020. Emerg. Microbes Infect. 11, 1508–1517. doi: 10.1080/22221751.2022.2078228 35582916 PMC9176688

[B31] Wang Y. G. W. Wang W. Wang Y. (2016). Significance of molecular switch-based detection of mutations to prediction of antimicrobial susceptibility of *Mycoplasma pneumoniae* . Chin. J. Microecology 28, 926–929. doi: 10.13381/j.cnki.cjm.201608012

[B32] Wang Y. Xu B. Wu X. Yin Q. Wang Y. Li J.. (2021). Increased macrolide resistance rate of M3562 *Mycoplasma pneumoniae* correlated with macrolide usage and genotype shifting. Front. Cell Infect. Microbiol. 11, 675466. doi: 10.3389/fcimb.2021.675466 34055671 PMC8149950

[B33] Wei W. Wang X. F. Liu J. P. Shen K. L. Ma R. Cui Z. Z.. (2019). Status of antibiotic use in hospitalized children with community-acquired pneumonia in multiple regions of China. China J. Contemp Pediatr. 21 (1), 11–17. doi: 10.7499/j.issn.1008-8830.2019.01.003 PMC7390181 30675857

[B34] Wu T. H. Wang N. M. Liu F. C. Pan H. H. Huang F. L. Fang Y. P.. (2021). Macrolide resistance, clinical features, and cytokine profiles in Taiwanese children with *Mycoplasma pneumoniae* infection. Open Forum Infect. Dis. 8, ofab416. doi: 10.1093/ofid/ofab416 34557557 PMC8454522

[B35] Xiao L. Ratliff A. E. Crabb D. M. Mixon E. Qin X. Selvarangan R.. (2020). Molecular characterization of *Mycoplasma pneumoniae* isolates in the united states from 2012 to 2018. J. Clin. Microbiol. 58, e00710–20. doi: 10.1128/JCM.00710-20 32817226 PMC7512161

[B36] Xin D. Mi Z. Han X. Qin L. Li J. Wei T.. (2009). Molecular mechanisms of macrolide resistance in clinical isolates of *Mycoplasma pneumoniae* from China. Antimicrob. Agents Chemother. 53, 2158–2159. doi: 10.1128/AAC.01563-08 19273685 PMC2681523

[B37] Yang T. I. Chang T. H. Lu C. Y. Chen J. M. Lee P. I. Huang L. M.. (2019). *Mycoplasma pneumoniae* in pediatric patients: do macrolide-resistance and/or delayed treatment matter? J. Microbiol. Immunol. Infect. 52, 329–335. doi: 10.1016/j.jmii.2018.09.009 30341022

[B38] Zheng Y. Hua L. Zhao Q. Li M. Huang M. Zhou Y.. (2021). The level of d-dimer is positively correlated with the severity of *Mycoplasma pneumoniae* pneumonia in children. Front. Cell Infect. Microbiol. 11, 687391. doi: 10.3389/fcimb.2021.687391 34336714 PMC8319762

[B39] Zhou Z. Li X. Chen X. Luo F. Pan C. Zheng X.. (2015). Macrolide-resistant *Mycoplasma pneumoniae* in adults in zhejiang, China. Antimicrob. Agents Chemother. 59, 1048–1051. doi: 10.1128/AAC.04308-14 25451048 PMC4335877

